# Does health voucher intervention increase antenatal consultations and skilled birth attendances in Cameroon? Results from an interrupted time series analysis

**DOI:** 10.1186/s12913-024-10962-9

**Published:** 2024-05-08

**Authors:** Isidore Sieleunou, Roland Pascal Enok Bonong

**Affiliations:** 1 The Global Financing Facility (GFF), Dakar, Senegal; 2 Research for Development International, 30883 Yaoundé, Cameroon

**Keywords:** Voucher, Results-based financing, Health financing, Antenatal consultation, Skilled birth attendance, Cameroon

## Abstract

**Background:**

Limited access to health services during the antenatal period and during childbirth, due to financial barriers, is an obstacle to reducing maternal and child mortality. To improve the use of health services in the three regions of Cameroon, which have the worst reproductive, maternal, neonatal, child and adolescent health indicators, a health voucher project aiming to reduce financial barriers has been progressively implemented since 2015 in these three regions. Our research aimed to assess the impact of the voucher scheme on first antenatal consultation (ANC) and skilled birth attendance (SBA).

**Methods:**

Routine aggregated data by month over the period January 2013 to May 2018 for each of the 33 and 37 health facilities included in the study sample were used to measure the effect of the voucher project on the first ANC and SBA, respectively. We estimated changes attributable to the intervention in terms of the levels of outcome indicators immediately after the start of the project and over time using an interrupted time series regression. A meta-analysis was used to obtain the overall estimates.

**Results:**

Overall, the voucher project contributed to an immediate and statistically significant increase, one month after the start of the project, in the monthly number of ANCs (by 26%) and the monthly number of SBAs (by 57%). Compared to the period before the start of the project, a statistically significant monthly increase was observed during the project implementation for SBAs but not for the first ANCs. The results at the level of health facilities (HFs) were mixed. Some HFs experienced an improvement, while others were faced with the status quo or a decrease.

**Conclusions:**

Unlike SBAs, the voucher project in Cameroon had mixed results in improving first ANCs. These limited effects were likely the consequence of poor design and implementation challenges.

**Supplementary Information:**

The online version contains supplementary material available at 10.1186/s12913-024-10962-9.

## Background

Reducing maternal, newborn, and child mortality is one of the world's top public health priorities. The third of the seventeen Sustainable Development Goals (SDGs) reflects the international commitment to improving maternal and child health. By 2030, the goals include reducing the global maternal mortality ratio to less than 70 per 100,000 live births, neonatal mortality to 12 per 1,000 live births at most, and under-five mortality to less than 25 per 1,000 live births [1].

However, despite considerable improvements in recent decades, maternal mortality has remained a major public health concern globally, with more than 295,000 maternal deaths in 2017 and sub-Saharan Africa (SSA) alone accounting for approximately 66% of this global picture [2]. On the other hand, despite dramatic reductions in child mortality over the last 30 years, the global burden of child deaths has remained immense, with a total of 5.2 million under-five deaths in 2019, representing an average of 14,000 deaths every day [3].

While from 2000 to 2017, the global maternal mortality ratio (MMR) decreased by 38% [2], Cameroon's MMR skyrocketed from 511 in 1998 to 782 in 2011 before declining to 467 in 2018 [4].

A priority toward ending preventable maternal and child deaths is to improve access to and use of quality health services and qualified nurses at birth [5, 6]. One of the basic elements is the presence of pregnant women at antenatal consultations. Previous studies have shown that performing prenatal consultations reduces the risk of neonatal mortality [7, 8].

However, women in developing countries encounter significant barriers to accessing conventional health services, including poor education, physical and financial barriers, and limited voice and decision-making power [9, 10]. The poor quality of available health services offers a further disincentive [6]. This translates to only half of parturient women receiving skilled assistance at delivery and many fewer receiving postpartum cares [6].

In Cameroon, the country’s comparatively slow reduction in maternal and child mortalities is likely due to insufficient coverage of reproductive, maternal, neonatal, child and adolescent health (RMNCAH) services; for instance, in 2018, an estimated 65% of women in Cameroon attended at least four antenatal consultations (ANC) visits, 69% gave birth with the assistance of qualified personnel, and 59% received postnatal care (PNC) [11]. In addition, these general estimates hide enormous disparities. Overall, 65% of the pregnant women who attended the four ANCs included more than 79% of those in urban areas but only 52% of those in rural areas. Moreover, while this rate was 91% in the richest quintile, only one-third (37%) of the poorest pregnant women attended the four ANCs [11].

The complexity of barriers to accessing care in developing countries indicates that any solution to improving maternal health service utilization must be comprehensive and address both supply- and demand-side health system constraints. This is particularly important in a context such as Cameroon where household out-of-pocket (OOP) spending was the single largest source of financing for the health sector, at 71 percent of total health spending in 2017, well above the WHO benchmark of 15-20 percent, and exceeding the average for SSA (33 percent) and countries of similar income such as Kenya (24 percent) and Ghana (40 percent) [12].

As ability to pay remains an important determinant of women’s access to healthcare, many countries have sought to improve coverage of maternal services by reducing financial barriers to seeking services [13, 14]. Strategies implemented at the country level include national health insurance and user fee removals/exemptions, and at the subnational level, community-based health insurance, health vouchers and conditional cash transfers [15].

Given that limited access to emergency obstetric and neonatal care (EmONC) is a major contributor to high maternal mortality [16], increasing pregnant women's use of health facilities for assisted delivery could help reduce maternal and new born morbidity and mortality, as previous studies have indicated [17, 18].

In recent years, there has been growing interest in the use of vouchers and other innovative financing mechanisms to increase access to EmONCs for low-income women [19–25]. By providing a financial or in-kind reward conditioned on the achievement of agreed-upon performance goals, vouchers are described as a promising holistic approach to foster the use of cost-effective services by the poor and other disadvantaged populations [22].

Vouchers can act on the demand side, the supply side, or both sides. Demand-side incentives encourage service use not only by reducing the financial burden but also by offering women a choice of providers and informing them of the benefits of using maternal health services. Supply-side incentives aim to improve the quality and responsiveness of service delivery.

To date, findings from the few assessments of reproductive health voucher programs suggest that, if implemented well, they have the potential to improve both assisted and facility-based deliveries [19, 20, 22, 24, 26]. Yet, there is a paucity of evidence based on rigorous evaluation studies, making it challenging to draw consistent conclusions about the impact of voucher initiatives and to make subsequent policy recommendations.

The current study evaluated a pilot voucher program in Cameroon, a country where approximately 39% of all deliveries took place at home at the time of the program’s inception [27]. The research aimed to assess the impact of the voucher scheme on first antenatal consultation and skilled birth attendance (SBA). In the following, we present a brief description of the Cameroon voucher program. We then present our data and methods, followed by the results. We end with a discussion of the study’s results, as well as the implications of these findings.

### Voucher program in Cameroon

Results from the 2014 Multiple Indicator Cluster Survey (MICS) indicate an enormous disparity in health outcomes among Cameroon's ten regions, with the three northern regions (Adamawa, North, Far North) bearing the brunt of the disease burden [27]. For example, while the Far North and North regions represented 27.5% of the total population of children under five years in 2014, both regions accounted for 63% of the total excess mortality during the same period [27]. In addition, while 65% of women nationwide gave birth with the help of qualified personnel, only 29%, 36% and 53% in the Far North, North and Adamawa regions, respectively, gave birth in the same conditions. Moreover, these three regions featuring the lowest frequencies of ANCs and assisted deliveries, were home to more than 60% of the country’s poorest population [28].

Initiated in 2015, the voucher programme is a government programme, supported with funding from German and French partners, that aims to reduce financial barriers to maternal and neonatal care in the three northern regions of Cameroon.

Under the project, (poor) women can purchase subsidized vouchers for 6000 FCFA (≈$11), a co-payment of 10% of the actual cost of the service package estimated at 60,000 FCFA (approximately USD109), that covered the cost of a benefit package including services for pregnant women and their new-borns up to 42 days after delivery. In addition, beneficiaries are provided with transportation from their house to the nearest health facility and transportation from health centers to referral hospitals. Health facilities offering services for the voucher scheme are compensated for extra costs incurred. All pregnant women living within the 3 northern regions of Cameroon were eligible for the programme. To be included in the programme, health facilities are required to meet minimum quality standards based on national guidelines for the provision of maternal care. Women can redeem vouchers at any participating facility, and the contracted facilities submit claims to be reimbursed at standard rates for each service provided.

At its inception, the programme implementation was outsourced to the ‘Centre International de Développement et de Recherche’ (CIDR), an international organization. Since November 2018, the management of the scheme has been transferred to a national entity: the Regional Funds for Health Promotion (RFHP). A transfer protocol signed between the ministry of public health (MPH) and CIDR made provisions for the training of the RFHP personnel to take over the implementation.

## Materials

### Study design, data source and study sample

To achieve the study objectives, we used a quasi-experimental study design. Specifically, for each health facility (HF) that was enrolled in the health voucher project, the potential effect of the project was measured using an analysis of interrupted time series [29–32]. This method compares changes in the indicators of interest before and after the start of the intervention. It is based on the fundamental assumption that, in the absence of intervention, the trend of the interest indicator remains unchanged over time [31]. It is desirable to have at least 12 observation points for the indicator or variable of interest before and after the start of the intervention, respectively [29].

We used secondary data from the monitoring and evaluation system database populated by the three regional implementing agencies of the health voucher project, let by the CIDR-CARE prior to the transfer of the project to the RFHP that began in 2018. These databases were updated quarterly by trained research assistants after monthly data collection from the registries of all health facilities enrolled in the project. Data quality control was carried out jointly by the team from the MPH in charge of monitoring project implementation and by the project team. The data used in this study are monthly aggregates of the variables of interest over the period from January 2013 to May 2018 (i.e., 65 months of observation).

The database contains information on 42 health facilities (HFs) enrolled in the health voucher project, spread across three regions: 12 HFs in the Adamawa region, 15 in the North region and 14 in the Far North region. These HFs were sequentially enrolled in the health voucher project and not at the same time. In the Adamawa region, activities started in 9 HFs in May 2015 and in 3 HFs in March 2016. In the North region, the implementation of activities started in May 2015 in one HF, in June 2015 in 5 HFs and in July 2016 in 9 HFs. For the Far North region, the intervention started in 4 HFs in June 2015, in 3 HFs in March 2016 and in 7 HFs in July 2016. For the analysis of each outcome, HFs included in the sample were those with at least 90% data completeness over the selected period. Thus, the sample sizes for analysis of the outcomes associated with the first antenatal consultation and assisted deliveries were 33 and 37, respectively.

### Study variables

#### Outcomes

Two dependent variables were considered for this evaluation: (i) the monthly number of first ANC visits in each HF and (ii) the monthly number of SBA in each HF.

#### Covariables

o X_it_: a time-dependent dichotomous variable that takes the value 0 for the months before the start of the health voucher project in HF i and 1 after the start of the project.
o T_t_: time variable measured in months, with values ranging from 0 (January 2013) to 64 (May 2018).
o X_it_*(T_t_-θ_i_): interaction variable between the variables X_it_ and T_t_ centered on the value corresponding to the month of project start in HF i (θ_i_).

### Statistical analysis

#### Descriptive analysis

To explore the outcomes, we used descriptive statistics (mean, median, standard deviation, interquartile range, absolute frequency, relative frequency) and trend curves.

#### Statistical modeling

For each HF and for each outcome, the estimation of the effects of the health voucher project was carried out using a negative binomial regression.

Since both outcomes are count variables, the choice of negative binomial regression instead of Poisson regression, which is the classic model for this type of variable, was considered to overcome the violation of the fundamental assumption underlying Poisson regression, which states that the mean is equal to the variance. Let Y_it_ be the value of the considered outcome observed in HF i at time t. Y_it_ follows a Poisson distribution with parameter μ_it_ (Y_it_ ~Poisson (μ_it_)). The general equation of the model used is shown below:

1 $$\log\ (\upmu_{\text{it}}) = \upbeta_{0} + \upbeta_{1} \text{T}_{\text{t}} + \upbeta_{2}\text{X}_{\text{it}} + \upbeta_{3}\text{X}_{\text{it}} \ast (\text{T}_{\text{t}} - \uptheta_{\text{i}}) + \upgamma_{\text{it}}$$

The other parameters of the model are described below.

- β_0_= intercept (value of the dependent variable at month 1 of follow-up);
- β_1_= slope of the outcome trajectory before the start of the health voucher project;
- β_2_= change in the level of outcome at the end of the first month of implementation of the health voucher project;
- β_3_= difference between the slope of the outcome trajectory after and before the start of the health voucher project;
- variable γ_it_ is the term that differentiates Poisson regression from negative binomial regression. In other words, e^γit^ follows a gamma distribution with mean 1 and variance α (e^γit^ ~ gamma (1/α, α)), with α being the overdispersion parameter.

The coefficient β_2_ assesses the immediate effect of the project and β_3_ assesses the effect of the project over time.

The graphs used to explore the evolution of outcomes over time highlighted the presence of seasonality. Thus, 11 dichotomous variables were considered in the different models. Equation (1) becomes log (μ_it_) = β_0_ + β_1_T_t_ + β_2_X_it_ + β_3_ X_it_*(T_t_-θ_i_) + ɸ_1_February + ɸ_2_March + ɸ_3_April + ɸ_4_May + ɸ_5_June + ɸ_6_July + ɸ_7_August + ɸ_8_September + ɸ_9_October + ɸ_10_November + ɸ_11_December + γ_it_.

The variables February, March … December take the value 1 if the observation relates to this month and 0 otherwise. The month of January was considered a reference.

Because the project did not start at the same time in all HFs, to obtain estimates representing the overall situation, a meta-analysis was used [33]. Thus, the pooled estimates and their confidence intervals were obtained by combining the regression coefficients of each HF using the inverse variance method. Random effects models were used to consider the strong heterogeneity highlighted by the statistics I^2^=100*(Q-df)/Q (with Q the statistics of Cochran's Q-test of heterogeneity and df the number of degrees of freedom corresponding here to the number of HFs minus one). The values 0%, 25%, 50% and 75% of the I^2^ statistics represent the following levels of heterogeneity: absent, weak, moderate, and strong, respectively [33, 34]. The incidence-rate ratio (IRR) for each HF per month as well as the aggregate estimates were graphically represented using a "forest plot". The analysis was stratified by region.

The statistical significance threshold used for interpreting the results was 5%. All the statistical analyses were performed with Stata/SE software version 14.2.

## Results

### Descriptive statistics

The results in Table 1 show that the overall level of data completeness is 98.9% for the monthly number of first ANC visits and 99.3% for the monthly number of SBAs. In all regions, better data completeness was observed in the post-start period of the intervention. For the descriptive statistics of the two variables of interest, overall, the average (respectively the median) of the monthly number of first ANC visits was 58.6 (respectively 50.0). For the monthly number of SBAs, the mean and median were 52.3 and 31.0, respectively. The observed differences between the means and medians illustrate the asymmetry of the distributions of these variables. We also found that the means and medians of these two variables appeared to be greater during the implementation period of the project than during the period prior to the intervention.

**Table 1:** Completeness of data and descriptive statistics of outcomes by region, before and during project implementation

	Total	Period	
		Before the project	During the project
ALL THREE REGIONS			
First antenatal consultations			
Number (%) complete observations	2066 (98.9%)	1067 (97.9%)	999 (100.0%)
Monthly average (SD)	58.6 (41.5)	54.7 (42.6)	62.7 (39.9)
Monthly median (IQR)	50.0 (28.0, 77.0)	44.0 (24.0, 70.0)	56.0 (32.0, 81.0)
Assisted deliveries			
Number (%) complete observations	2333 (99.3%)	1192 (98.8%)	1141 (99.9%)
Monthly average (SD)	52.3 (61.2)	42.0 (60.6)	63.1 (60.1)
Monthly median (IQR)	31.0 (15.0, 63.0)	18.0 (10.0, 46.0)	42.0 (25.0, 74.0)
ADAMAWA			
First antenatal consultations			
Number (%) complete observations	614 (98.7%)	274 (97.2%)	340 (100.0%)
Monthly average (SD)	60.5 (48.7)	52.0 (53.4)	67.4 (43.6)
Monthly median (IQR)	43.0 (25.0, 87.0)	29.5 (15.0, 59.0)	56.0 (36.0, 88.0)
Assisted deliveries			
Number (%) complete observations	809 (99.0%)	359 (98.1%)	450 (99.8%)
Monthly average (SD)	63.2 (81.1)	52.6 (86.3)	71.6 (75.8)
Monthly median (IQR)	28.0 (13.0, 77.0)	14.0 (9.0, 36.0)	40.0 (24.0, 81.0)
FAR NORTH			
First antenatal consultations			
Number (%) complete observations	643 (98.9%)	362 (98.1%)	281 (100.0%)
Monthly average (SD)	56.3 (41.1)	53.0 (38.9)	60.6 (43.3)
Monthly median (IQR)	51.0 (23.0, 74.0)	48.0 (21.0, 72.0)	54.0 (27.0, 79.0)
Assisted deliveries			
Number (%) complete observations	712 (99.6%)	399 (99.3%)	313 (100.0%)
Monthly average (SD)	46.8 (49.4)	35.6 (42.7)	61.0 (53.7)
Monthly median (IQR)	27.0 (14.0, 58.0)	17.0 (11.0, 37.0)	39.0 (26.0, 71.0)
NORTH			
First antenatal consultations			
Number (%) complete observations	809 (99.0%)	431 (98.2%)	378 (100.0%)
Monthly average (SD)	59.0 (35.4)	57.9 (37.4)	60.2 (32.9)
Monthly median (IQR)	53.0 (33.0, 75.0)	50.0 (33.0, 71.0)	56.0 (34.0, 78.0)
Assisted deliveries			
Number (%) complete observations	812 (99.4%)	434 (98.9%)	378 (100.0%)
Monthly average (SD)	46.3 (43.7)	39.1 (46.1)	54.7 (39.1)
Monthly median (IQR)	37.0 (17.0, 59.0)	25.0 (12.0, 50.0)	47.5 (29.0, 68.0)

*SD* Standard deviation, *IQR* Interquartile range

**Fig. 1:**
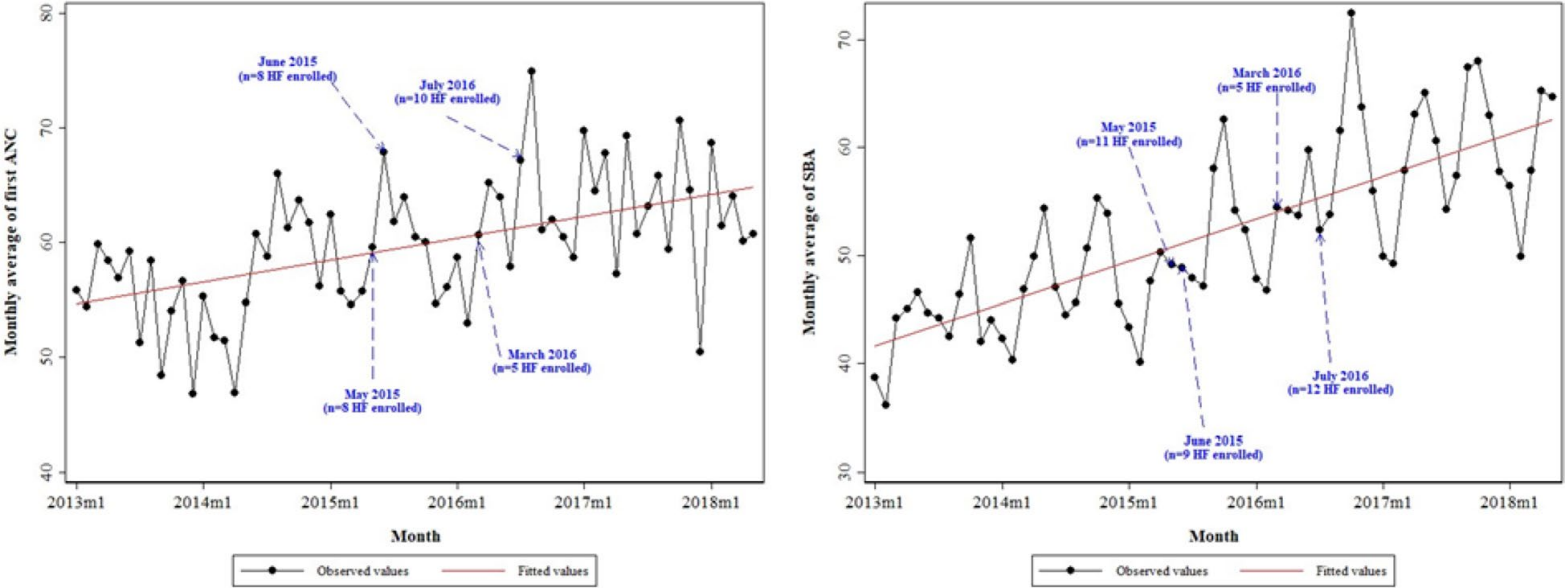
Evolution of the monthly averages of the number of first ANC visits and SBAs in the selected Health facilities between January 2013 and May 2018

Furthermore, Fig. 1 shows that there was an increasing trend over time for the monthly average of the first ANC and the monthly average of the SBA. It also emerged that the positive slope was more abrupt for SBA.

### Effects of the health voucher project

#### First antenatal consultation (ANC)

Table 2 and Figure S3 displays contrasting results. Overall, at the end of the first month of implementation of the project, controlling for other variables, a statistically significant increase of nearly 26% in the monthly number of first ANCs was observed in the 33 HFs considered in the study sample (IRR = 1.258 [95% CI: 1.075, 1.472]). A similar increase was recorded in the North region but was not statistically significant (IRR = 1.246 [95% CI: 0.976, 1.591]). In the Adamawa region, the increase was nearly 73% (IRR = 1.726 [95% CI: 1.117, 2.668]). Conversely, in the Far North region, a nonsignificant reduction of 0.2% was noted (IRR = 0.998 [95% CI: 0.882, 1.129]). These overall results hid disparities across facilities. In the Adamawa region, out of 10 HFs, there was a statistically significant increase in the monthly number of first ANCs at the end of the first month of project implementation in five HFs and a statistically significant decrease in one HF. In the Far North region, of the 10 HFs, a statistically significant increase was recorded in two HFs, and a statistically significant reduction was recorded in one HF. In the North region, of the 13 HFs, six exhibited a statistically significant increase in the aforementioned indicator and one exhibited a statistically significant decrease.

**Table 2:** Pooled regression coefficients obtained using Inverse-variance weighted random-effects meta-analysis of negative binomial regression coefficients estimated for each health facility, stratified by region

		**Overall**		**Adamawa**		**Far North**		**North**	
		**Coefficient (95% CI)**	**I**^**2**^	**Coefficient (95% CI)**	**I**^**2**^	**Coefficient (95% CI)**	**I**^**2**^	**Coefficient (95% CI)**	**I**^**2**^
**Outcome**: log of monthly number of first ANC	**β**_**0**_	3.783 (3.543, 4.022)	97.5%	3.562 (2.945, 4.179)	98.8%	3.711 (3.282, 4.140)	97.5%	4.018 (3.772, 4.264)	93.2%
	**β**_**1**_	0.000 (-0.003, 0.004)	85.4%	0.002 (-0.003, 0.007)	59.1%	0.006 (0.002, 0.009)	62.6%	-0.005 (-0.013, 0.003)	90.7%
	**β**_**2**_	0.230 (0.072, 0.387)	93.3%	0.546 (0.111, 0.981)	96.6%	-0.002 (-0.126, 0.121)	69.4%	0.220 (-0.024, 0.464)	91.8%
	**β**_**3**_	-0.004 (-0.009, 0.002)	82.1%	-0.001 (-0.010, 0.008)	78.2%	-0.008 (-0.017, 0.001)	83.2%	-0.003 (-0.013, 0.008)	85.0%
**Outcome**: log of monthly number of SBA	**β**_**0**_	3.037 (2.689, 3.385)	99.2%	3.020 (2.269, 3.772)	99.5%	3.057 (2.389, 3.725)	99.2%	3.042 (2.595, 3.489)	98.7%
	**β**_**1**_	0.001 (-0.002, 0.005)	87.7%	0.005 (-0.001, 0.011)	82.3%	-0.002 (-0.008, 0.004)	88.8%	0.001 (-0.005, 0.007)	88.9%
	**β**_**2**_	0.448 (0.306, 0.591)	93.9%	0.543 (0.276, 0.810)	94.1%	0.402 (0.115, 0.689)	95.8%	0.394 (0.172, 0.617)	91.9%
	**β**_**3**_	0.009 (0.002, 0.016)	92.1%	0.007 (-0.009, 0.023)	95.2%	0.016 (0.004, 0.028)	92.4%	0.004 (-0.004, 0.012)	79.1%

Moreover, regarding the difference between the slope of the trajectory of the first ANC after and before the start of the project, Table 2 and Figure S4 does not show statistically significant results, either overall or by region. However, in one HF in the Adamawa region, a statistically significant increase in the slope of the trajectory of the first ANC was observed during the project implementation period compared to the situation prior to the intervention. Conversely, a statistically significant decrease was recorded in one HF. In the Far North region, no HF exhibited a statistically significant increase, but a statistically significant decrease was observed in two HFs. In the North region, two HFs exhibited a statistically significant increase, and five HFs exhibited a statistically significant decrease.

#### Skilled birth attendance (SBA)

Table 2 and Figure S7 shows that by the end of the first month of implementation of the project, a statistically significant increase of nearly 57% in the monthly number of SBAs was recorded in the 37 HFs selected in the study sample, controlling for other variables (IRR = 1.566 [95% CI: 1.358, 1.806]). 

A statistically significant increase in this indicator was also observed in each of the three regions. However, there were disparities between HFs. In the Adamawa region, out of 13 health facilities, there was a statistically significant increase in the monthly number of assisted deliveries at the end of the first month of project implementation in nine HFs and a statistically significant decrease in one HF. In the Far North region, of the 11 HFs, a statistically significant increase was recorded in eight HFs and a statistically significant decrease was recorded in two HFs. In the North region, of the 13 HFs, seven recorded a statistically significant increase and one a statistically significant decrease in the indicator of interest.

In addition, Table 2 and Figure S8 indicates that, overall, the intervention had a positive effect on SBAs (IRR = 1.009 [95% CI: 1.002, 1.016]). A similar finding is observed in the three regions, with the Far North region being the only region that was statistically significant. When considering the analysis of HFs, the results are mixed. In the Adamawa region, a positive and statistically significant result was recorded for four HFs while a negative and statistically significant result was observed for three HFs. In the Far North region, statistically significant results were recorded for five HFs and all these results were positive. In the North region, two HFs recorded a positive result and three recorded a negative result.

The high values of the I^2^ statistics reveal that a very large proportion of the total observed variance is due to a real difference in effect measures between HFs (Figure S1 to S8).

## Discussion

Our study explored the effect of the Health voucher Project on the use of health services. Overall, a statistically significant increase was observed in the number of first ANCs at the end of the first month of project implementation (success). However, this improvement was not sustained over time, with less than 10% of all HFs (3/33) experiencing an increase in ANCs.

For the SBAs, there was a statistically significant increase at the end of the first month of project implementation, with a sustained pattern over time. When looking at the individual HFs, 2/3 (65%) recorded success at the end of the first month of implementation, while 30% experienced overall improvement during the project implementation compared to the period before the start of the project.

These findings suggest that between the pre-intervention/roll-out and full implementation phases, the Cameroon voucher programme modestly increased the use of facility for ANC and SBA, consistent with previously reported results from evaluations of maternal health voucher programmes from other LMICs [21, 35–38].

Our results therefore indicate that in a country such as Cameroon, where progress toward universal health coverage is still to be achieved [39], reducing financial risk by providing subsidies to offset the costs of receiving RMNCAH services may be a good cost-effective intervention to improve service utilization.

Pregnant women were more likely to use the voucher system for SBAs than for the first ANC visits. One explanation could be the late attendance of pregnant women at health facilities, as more than 70% of pregnant women in these three regions are reported to have their first contact with a health facility after the first trimester of pregnancy [27], or the late acquisition of vouchers. In-depth discussions with health care providers and direct beneficiaries are needed to better understand the realities underlying these trends.

The decrease in first ANC and SBA over time in some HFs could be explained by the increasing expansion of service coverage, with the opening of new health facilities that were not yet included in the project and that were used by some pregnant women. On the other hand, the context of growing insecurity linked to Boko Haram and other rebel groups in neighboring countries could also constitute a barrier to the use of health facilities in these regions.

It is also important to note that the voucher program is conceptually designed to target the poorest populations. In Cameroon, however, the project covers all women of reproductive age in the intervention areas, regardless of socioeconomic status. We suspect that the contribution of the 6,000 FCFA ($11 US) remains a major barrier to the use of health services for the poorest women, especially since the project covers mostly urban areas, raising the question of program equity as reported elsewhere [13, 14, 16]. This challenge was also highlighted in an unpublished qualitative study.

Focusing on strategies that prioritize the poorest women and strengthen community engagement can ensure equity and achieve sustainable results over time. For example, in Bangladesh and Cambodia, the voucher programme focused on those most in need and reimbursed care givers in facilities to motivate them [40, 41]. Moreover, both countries have successfully partnered with recipient communities to improve the targeting of the poor [40–42].

In addition to stimulating demand, voucher schemes are often proposed as a way to improve the quality of care, as is the case in Cameroon, where health facility accreditation mechanisms are used, alongside the performance-based financing scheme implemented nationwide. However, experiences show that providers may find reimbursement rates to be unattractive and engage in practices such as providing inconsistent quality of care or ‘skimming’ programme users who require minimal intervention. Moreover, as reported in other voucher programs, the most significant problem faced by the voucher scheme in Cameroon was the delay in paying for health facilities, which led to staff demotivation and mistrust between the managers of the scheme and the beneficiaries [41] and suggested a need for greater attention to issues related to implementation in such a program [26].

This study helps to extend the body of knowledge generated by previous research on health voucher programmes in LMICs. However, in interpreting our findings, the strengths and limitations of the study design should be considered.

First, most studies on voucher programmes to date have examined the immediate or shorter-term impact of the intervention on service utilization [21]. Our study examined the immediate to longer-term effects of the intervention and used a quasi-experimental design, known as a reliable approach, to provide robust estimates of the effect of an intervention when a randomized controlled trial cannot be conducted or when a control group is lacking [29, 31]. Unlike in cross-sectional observational studies, interrupted time series analysis allows us to estimate the dynamics of change driven by the intervention, controlling for secular changes that might have occurred in the absence of the intervention [29, 43]. This approach thus makes it possible to observe whether the intervention has an immediate or delayed, sudden or gradual effect and whether this effect persists or is temporary. Furthermore, there is no real consensus on the number of observation points needed to use the interrupted time series method. However, the statistical power increases with the number of time points [30]. Some authors recommend 12 observation points before and after the start of the intervention [29]. In our study, only one HF had 10 observation points before the start of the project, and the others had observation points ranging from 14 and 42. During the project implementation period, the number of observations varied between 23 and 37.

At the time of the study, 81 facilities had already enrolled in the voucher project. We limited ourselves to 33 HFs for the first ANC and to 37 HFs for the SBA analysis because the data prior to the project were either unavailable or insufficient. Therefore, the results presented in this study may be a fragmented view of the project’s effect. In addition, analysis that could provide insight into the RMNCAH continuum of care was not possible due to the limited quality of data (high frequency of missing data) for some key indicators, such as the fourth ANC and postnatal consultation, as reported with other voucher programs [22, 44–46].

In identifying the impact of an intervention, it is important that there are no exogenous factors influencing the results. During the implementation of the voucher program in Cameroon, there were no closures of health facilities that could have an impact on the two selected indicators. Population growth naturally leads to an increase in the number of pregnant women in absolute terms, and consequently to an increase in the number of SBAs. Because demographic data were only available for each health district and not for each health facility, estimates of expected populations or pregnant women were not included into the various negative binomial regression models as a control variable. As a result, the estimates obtained may be biased.

It is also important to point out that due to its fragility, the northern part of the country is a convergence zone of several programs and projects, including those of health. Therefore, other interventions may have also contributed to the achievement of these outcome levels. One of the most important programmes is the National Multi-sector Program to Combat Maternal, Newborn and Child Mortality, which was created in 2013.

Finally, we would like to underline that the fidelity of the program's implementation was hampered by deviations, leading for instance to extending the intervention to all women of childbearing age. At present, the program is more akin to an obstetric risk insurance system, as described for example in Mauritania [47].

## Conclusion

This study provided important insight into the Cameroon voucher scheme. The intervention had a significant early effect on the first ANC and SBA but failed to effectively sustain these results over time for the first indicator. These mixed effects were likely the consequence of poor design and implementation challenges, including the fact that the programme did not include specific equity measures to facilitate uptake by the poorest people. This suggests that for a complex intervention such as a voucher, it is critical to properly implement practice strategies that can sustain the long-term impact of the programme.

### Supplementary Information

**Supplementary Material 1.**

## Data Availability

The data that support the findings of this study are available from the Ministry of Public Health (MPH) of Cameroon, but restrictions apply due to the terms of our contract with the MPH, and so, data are not publicly available. The corresponding author should be contacted for the process to request data access.

## Abbreviations

ANC: Antenatal consultation
CIDR: Centre International de Développement et de Recherche
EmONC: Emergency obstetric and neonatal care
CFA: Communauté financière africaine
HF: Health facility
LMIC: Low- and middle-income country
MMR: Maternal mortality ratio
MICS: Multiple Indicator Cluster Survey
MPH: Ministry of public health
OOP: Out-of-pocket
PNC: Post-natal care
RFHP: Regional Funds for Health Promotion
RMNCAH: Reproductive, maternal, neonatal, child and adolescent health
SBA: Skilled birth attendance
SDG: Sustainable Development Goals
SSA: Sub Saharan Africa
USD: United States dollar
WHO: World health organization
