# Supplementary material for: Does health voucher intervention increase antenatal consultations and skilled birth attendances in Cameroon? Results from an interrupted time series analysis
Source: BMC Health Serv Res. 2024 May 8;24:602. doi: 10.1186/s12913-024-10962-9 (PMC11080306; doi:10.1186/s12913-024-10962-9)
Supplement: Supplementary file 1 — Supplementary Material 1. [file 12913_2024_10962_MOESM1_ESM.docx]

|  |
| --- |

Figure 2

Inverse-variance weighted random-effects meta-analysis of the slopes of the trajectory of the monthly number of first ANCs before the start of the programme (β_1_), stratified by region.

|  |
| --- |

Figure 3

Inverse-variance weighted random-effects meta-analysis of change in the level of the monthly number of first antenatal ANCs at the end of the first month of implementation of the programme (β_2_), stratified by region.

|  |
| --- |

Figure 4

Inverse-variance weighted random-effects meta-analysis of difference between the slope of the monthly number of first ANCs trajectory after and before the start of the programme (β_3_), stratified by region.

| 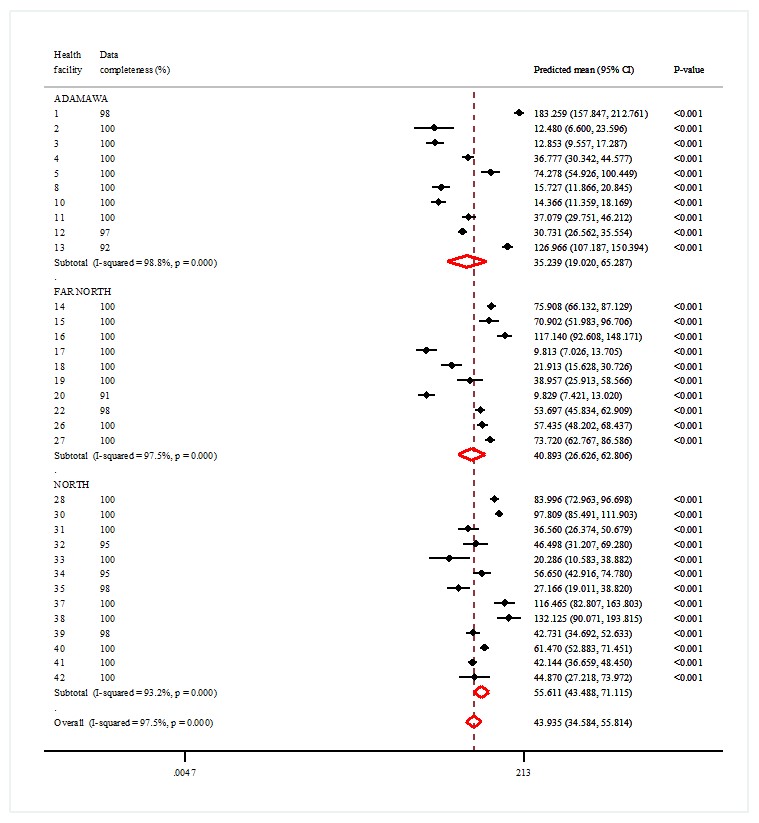 |
| --- |

Figure 5

Inverse-variance weighted random-effects meta-analysis of the predicted mean number of first ANC in the first month of the observation period (β_0_), stratified by region.

|  |
| --- |

Figure 6

Inverse-variance weighted random-effects meta-analysis of the slopes of the trajectory of the monthly number of SBAs before the start of the programme (β_1_), stratified by region.

|  |
| --- |

Figure 7

Inverse-variance weighted random-effects meta-analysis of change in the level of the monthly number of SBAs at the end of the first month of implementation of the programme (β_2_), stratified by region.

|  |
| --- |

Figure 8

Inverse-variance weighted random-effects meta-analysis of difference between the slope of the monthly number of SBAs trajectory after and before the start of the programme (β_3_), stratified by region.


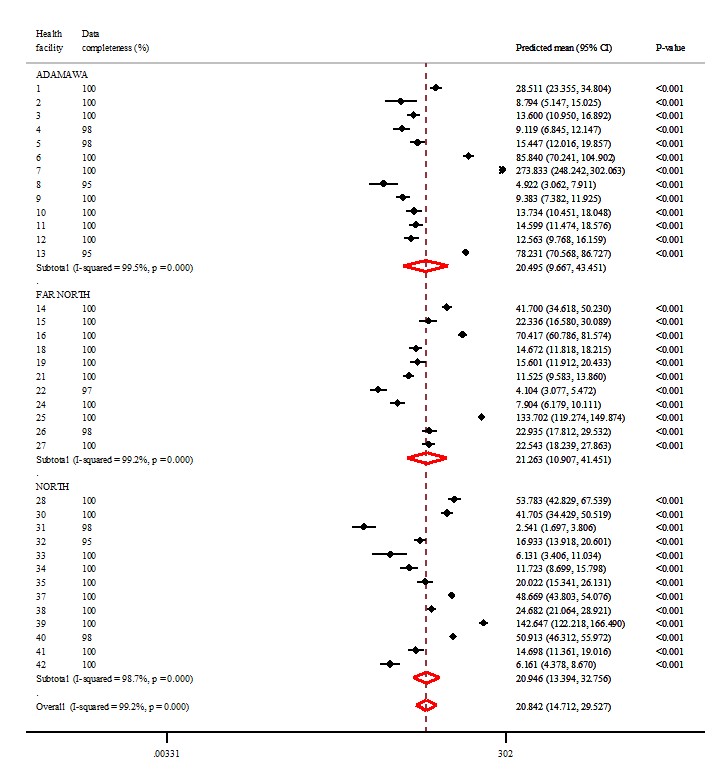


Figure 9

Figure: Inverse-variance weighted random-effects meta-analysis of the predicted mean number of SBA in the first month of the observation period (β_0_), stratified by region.
